# The effect of physical activity and body mass index on menopausal symptoms in Turkish women: a cross-sectional study in primary care

**DOI:** 10.1186/1472-6874-14-38

**Published:** 2014-03-06

**Authors:** Makbule Neslisah Tan, Mehtap Kartal, Dilek Guldal

**Affiliations:** 1 1st Family Health Center, Iğdır, Turkey; 2 Department of Family Medicine, Faculty of Medicine, Dokuz Eylul University, Izmir, Turkey

**Keywords:** Physical activity, Body mass index, Menopausal symptom

## Abstract

**Background:**

Considering the fact that, due to recent evidence, many women no longer prefer hormone replacement therapy, it is especially important to develop intervention options to alleviate menopausal symptoms. Although there is conflicting evidence concerning effectiveness, there is an indication that physical activity and weight control may be useful for alleviating symptoms. The aim of this study was to investigate the effect of physical activity and body mass index on menopausal symptoms among menopausal women in Turkey.

**Methods:**

305 women between the ages of 45 and 60 who visited the health center for various reasons were recruited into this cross-sectional study. Menopausal women, who visited one of five family physicians working in the same area, were included in the analyses. The Menopause Rating Scale, International Physical Activity Questionnaire and a generic medical and socio-demographic information questionnaire were used.

**Results:**

Women who were physically active had lower total menopausal (p < 0.001), somato-vegetative (p = 0.004), psychological (p = 0.002), and urogenital (p < 0.001) symptom scores than women who were less active. No differences in vasomotor symptoms were recorded related to physical activity level; significant differences were found for most menopausal symptoms, including sleep (p = 0.009) and sexual (p = 0.043) problems, joint and muscular discomfort (p < 0.001) and vaginal dryness (p = 0.016). BMI was not associated with total menopausal symptoms and with the subscales, excluding depressive mood (p = 0.009). A significant increasing trend in the rate of depressive mood was observed from normal through overweight to obese participants. The mean scores of the total menopausal symptoms were lower among the participants who were well educated, currently working and without chronic diseases.

**Conclusions:**

Physical activity may play an important role in alleviating menopausal symptoms. As part of preventive medicine, primary care physicians should also stress lifestyle changes, including physical activity, to manage menopausal symptoms.

## Background

The menopause is a significant event in most women’s lives and is related to ovarian failure and follicular atresia. It is characterized by the loss of ovarian function following the reduction in the secretion of estrogen, permanent cessation of menstruation and the loss of reproductive ability. It affects women’s health in biological, psychological and social aspects.

Menopause can lead to a wide range of symptoms including hot flashes, night sweats, sleeping problems, emotional and cognitive symptoms, irritability, anxiety, vaginal itching and dryness, and urinary symptoms. Reported hot flushes rates for perimenopausal women ranged from 40 to 60% [1]. The prevalence of vaginal atrophy in the early stages of the menopause increases as a woman advances through the postmenopausal years [2].

Although the nature and prevalence of menopausal symptoms are similar for most women, there are variations across and within cultures which are due to differences among lifestyle, socioeconomic status and the self-perception of individuals [3]. Menopausal symptoms may become problem not for only women, but also for their families, colleagues and communities. For this reason, clinicians who provide care during the menopause, have a significant opportunity for the provision of preventive medicine [4]. To fully benefit from this opportunity, physicians should be equipped with the means to alleviate the symptoms of the menopause.

The Global Consensus Statement on Menopausal Hormone Therapy’s recommendations in November 2012 [5] states, HRT is the most effective treatment for vasomotor symptoms and urogenital atrophy, but it has a complex pattern of risks and benefits. Current international consensus suggest that the hormonal treatment of menopause should be individualized and that the lowest dose of estrogen providing relief should be used for the shortest period of time in menopausal women. However, women may choose not to use hormone therapy because of possible adverse effects. While many women are now seeking alternatives, attention is increasingly directed toward non-hormonal approaches such as lifestyle changes to manage symptoms. There are promising results in this aspect as a result of lifestyle changes. The benefits of physical activity and weight control on health and wellbeing are well-known; however, the information concerning the explanation of the relationship between lifestyle changes and menopausal symptoms is very limited.

Evidence from studies concerning the effects of physical activity and exercise on vasomotor and other menopausal symptoms is conflicting. There are studies stating that physically active women have fewer menopausal complaints [6-9], or vice versa [10]. Despite the fact that many studies do not support a relation between physical activity and vasomotor symptoms [7,11-14], a recent study in Brasil, indicated that most of the women with no hot flashes were observed to be very active [9]. There are also studies indicating no sufficient evidence to determine the effectiveness of exercise as a treatment for vasomotor symptoms [15,16]. But also, Aiello et al. reported that the intervention group who underwent physical exercise experienced a significant increase in the severity of hot flashes [15]. A recent study suggested that moderate aerobic exercise decreases hot flashes; however, in women with lower fitness levels, more daily moderate physical activity leads to more self-reported symptoms [17].

There are conflicting results related to the effect of body mass index on menopausal symptoms, especially hot flashes. Several studies show that BMI is the main determinant of endogenous estrogen levels. In addition, it appears that estradiol (E1) and estrone (E2) are at higher levels among obese women than women within the normal weight range. Some studies demonstrate the fact that fewer vasomotor symptoms occur in obese women as compared to non-obese women [7,18]. In contrast, Klinga et al. reported that E1 and E2 levels decrease earlier in obese women as compared to non-obese women [19].

In addition, with regard to recent evidence, the impact of body mass index (BMI) and physical activity on menopausal symptoms is still unclear. The relationship between menopausal symptoms with physical activity and BMI may differ depending on the specific symptom and sociodemographic factors may have an effect on the symptoms. Most studies of menopause to date have been based either on Euro-American populations or far away from Turkey. To our knowledge, the effect of physical activity and body mass index on menopausal symptoms and associated sociodemographic characteristics have not been studied in Turkey. The objective of this study was to determine the effects of physical activity and BMI on menopausal symptoms in addition to sociodemographic factors influencing menopausal symptoms of Turkish women aged 45–60 years.

## Methods

This cross-sectional study was performed in primary care, between August and October 2009, in a semi-urban area of Bornova, a district of Izmir, Turkey. Menopausal women, who visited one of five family physicians who were working in the same area, were included in the analyses.

### Study sample and criteria

305 menopausal women between the ages of 45–60 who consulted the health center for any reason were recruited into this cross-sectional study. Women were asked questions related to their menstrual cycles in order to include appropriate participants who were in menopausal transition, either perimenopausal or postmenopausal period. Concerning menopausal status, definitions were defined according to the Stages of Reproductive Aging Workshop (STRAW) [20]: perimenopausal (change in menstrual cycle length of longer than 7 days/≥60 days amenorrhea/≥2 skipped cycles in the last 12 months) and postmenopausal (no more menses in the last 12 months). Exclusion criteria included: pregnancy or breastfeeding, hysterectomy with intact ovary, chronic irregular menstruation, use of hormonal medication in the last 3 months or the inability to complete the questionnaire. The present study was approved by the Dokuz Eylul University Medical Faculty Ethics Committee. Written informed consent for participation in the study was obtained from participants.

### Instrumentation and data collection

The study was described to the participants. Data were collected via face-to-face interviews. The demographic data collected in this study included socio-demographic (age, educational level, employment and marital status) and general health related (smoking, chronic disease, HRT usage) factors. Participants were asked to complete the Menopause Rating Scale (MRS) and International Physical Activity Questionnaire (IPAQ).

#### The Menopause Rating Scale-MRS

MRS was developed in 2000 by Heinmann et al. [21]. The Turkish version of MRS has been validated by Gürkan et al. in 2005 (the Cronbach α = 0.84) [22].

The MRS is a Likert-type scale which is composed of 11 items assessing menopausal symptoms divided into three subscales: somato-vegetative, psychological and urogenital. Each item is graded from 0 (not present) to 4 (1 = mild; 2 = moderate; 3 = severe; 4 = very severe). The total and psychological, somato-vegetative and urogenital subscale MRS scores that can be obtained at a minimum and maximum are 0–44; 0–16; 0–16; 0–12 respectively, and are graded according to the severity of symptoms as: not present, mild, moderate and severe. While the increase in the total score of the scale indicates an increase in the severity of the symptoms, a decreasing degree of severity of symptoms means an improvement of health-related Quality of Life in aging women [23].

#### International Physical Activity Questionnaire-IPAQ

The IPAQ was developed by the International Group for Consensus of Physical Activity Measurements and the Turkish version was validated by Öztürk (Test–retest reliability = 0.69) [24]. The IPAQ-short version, which includes seven items, is used to measure the frequency and duration of vigorous intensity, moderate-intensity, and walking-level physical activity for young and middle-aged adults (15–69 years). To calculate the weekly physical activity (MET-minute/ week), the number of minutes per week dedicated to each activity is multiplied by the specific MET score for that activity. The IPAQ sitting question is an additional indicator variable of time spent in sedentary activity and is not included as part of any summary score of physical activity [25]. Data from the questionnaire were gathered for each item (walking, moderate intensity, vigorous intensity) to estimate the total amount of time spent in physical activity per week. Three levels of physical activity were proposed at the end: low (no activity is reported or some activity is reported but not enough to meet categories moderate and high), moderate and high [24].

#### Body mass index-BMI

Height and weight were measured in light clothing and without shoes, using standardized instruments (Mewa Gmbtl/Schwerin-M318800). BMI was defined as weight in kilograms divided by the square of height in meters. The classification determined by the World Health Organization (WHO) was used to classify the participants accordingly [26].

### Statistical analysis

Data were analyzed using the SPSS software (version 15.0) package program. For descriptive and categorical variables, data were expressed as frequency and percent distribution; and continuous variables were presented as means ± standard deviation. In comparative analyses, Chi-square and Fisher’s exact test were used to compare categorical data. As normality assumptions were not satisfied for continuous variables they were analyzed by nonparametric methods as Mann Whitney-U, Kruskal Wallis, and Spearman’s correlation tests. Tukey was applied for multiple comparisons in post-hoc-tests. A p value less than 0.05 was considered statistically significant.

## Results

The demographic and health-related characteristics of the study population (n = 305) are reported in Table 1. The mean age was 52.1 ± 4.3 years. Based on the scores of IPAQ, 173 (56.7%) women were classified as having low level of physical activity. The mean BMI was determined 31.9 ± 5.8 (minimum: 16.0, maximum: 56.3).

**Table 1:** Sociodemographic and health-related characteristics (n = 305)

		**n**	**%**	**Mean ± SD (Range)**
**Age**	45–50	117	38.4	52.1 ± 4.3 (45–60)
	51–55	119	39.0	
	56–60	69	22.6	
**Educational level**	No formal education	42	13.8	
	Primary school	212	69.5	
	Above primary	51	16.7	
**Employment status**	Working	22	7.2	
	Non-working	177	58.0	
	Retired	106	34.8	
**Marital status**	Married	263	86.2	
	Divorced, widowed, single	42	13.8	
**Chronic disease**	Yes	178	58.4	
	No	127	41.6	
**Smoking status**	Ex/Current	109	35.7	
	Never	196	64.3	
**HRT usage**	Ex/Current	62	20.3	
	Never	243	79.7	
**Sitting time**				376.7 ± 111.9 (60–780)
**IPAQ**	Low	173	56.7	1205.2 ± 1639.9 (0–14400)
	Moderate	115	37.7	
	High	17	5.6	
**Body mass index**	≤24.9	27	8.8	31.9 ± 5.8 (16.0–56.3)
	25–29.9	84	27.5	
	≥30	194	63.6	

The mean somato-vegetative, psychological and urogenital subscale and total MRS scores were 4.6 ± 3.2, 4.7 ± 3.5, 2.9 ± 2.3 and 12.2 ± 7.2, respectively. The mean subscale and total MRS scores in relation to sociodemographic and health-related characteristics are reported in Table 2. The mean scores of total MRS score as well as each subscale of the MRS was determined to be significantly lower in women whose educational levels were above primary than primary school and women with no history of chronic disease. Somato-vegetative mean scores were found to be higher in non-working women than those retired. The MRS total scores were also higher in non-working participants. Urogenital subscale mean scores were found to be much higher in married women than women who were single. In women who have a history of HRT use, somato-vegetative subscale scores were found to be significantly higher compared to those who have never used HRT (p = 0.047). No other relation was observed between the MRS scores and other sociodemographic and health-related characteristics.

**Table 2:** Sociodemographic and health-related characteristics and comparisons of means of total and subscale scores

	Somato-vegetative*	Psychological*	Urogenital*	Total*
**MRS sores**	4.6 ± 3.2	4.7 ± 3.5	2.9 ± 2.3	12.2 ± 7.2
**Age**				
45–50	4.8 ± 3.2	5.0 ± 3.5	2.9 ± 2.4	12.6 ± 7.1
51–55	4.7 ± 3.3	4.6 ± 3.6	2.9 ± 2.2	12.3 ± 7.4
56–60	4.1 ± 2.9	4.3 ± 3.2	3.0 ± 2.4	11.4 ± 7.1
**p-values**	0.582	0.365	0.956	0.490
**Educational level**				
No formal education	4.4 ± 3.0	4.7 ± 3.5	2.5 ± 2.0	11.6 ± 6.1
Primary school	5.0 ± 3.3	4.9 ± 3.5	3.2 ± 2.3	13.1 ± 7.5
Above primary	3.2 ± 2.4	3.5 ± 2.9	2.2 ± 2.3	9.0 ± 5.9
**p-values**	*0.002*	*0.031*	*0.007*	*0.001*
**Employment status**				
Working	3.8 ± 2.2	3.9 ± 3.6	2.5 ± 2.3	10.2 ± 6.7
Non-working	5.1 ± 3.4	5.0 ± 3.4	3.1 ± 2.4	13.1 ± 7.5
Retired	4.0 ± 2.9	4.4 ± 3.4	2.8 ± 2.2	11.2 ± 6.7
**p-values**	*0.026*	0.094	0.473	*0.039*
**Marital status**				
Married	4.6 ± 3.1	4.6 ± 3.5	3.2 ± 2.3	12.4 **±** 7.2
Divorced/Separated, widowed, single	4.8 ± 3.7	5.1 ± 3.3	1.3 ± 1.4	11.1 ± 7.0
**p-values**	0.732	0.278	*<0.001*	0.464
**Chronic disease**				
Yes	5.0 ± 3.3	5.1 ± 3.5	3.2 ± 2.3	13.3 ± 7.4
No	4.0 ± 2.8	4.1 ± 3.3	2.6 ± 2.2	10.7 ± 6.6
**p-values**	*0.016*	*0.005*	*0.034*	*0.002*
**Smoking status**				
Ex/Current	5.1 ± 3.5	5.1 ± 3.6	2.9 ± 2.1	13.1 ± 7.5
Never	4.3 ± 2.9	4.5 ± 3.3	3.0 ± 2.4	11.7 ± 7.0
**p-values**	0.089	0.144	0.919	0.109
**HRT usage**				
Ex/Current	5.4 ± 3.5	5.1 ± 3.6	3.2 ± 2.3	13.7 ± 7.7
Never	4.4 ± 3.1	4.6 ± 3.4	2.9 ± 2.3	11.8 ± 7.0
**p-values**	*0.047*	0.299	0.236	0.118
**Physical activity level**				
Low	5.1 ± 3.2	5.1 ± 3.4	3.4 ± 2.4	13.6 ± 7.4
Moderate	4.1 ± 3.1	4.3 ± 3.5	2.3 ± 2.0	10.8 ± 6.6
High	3.3 ± 2.6	2.8 ± 3.1	2.0 ± 1.8	8.1 ± 6.5
**p-values**	*0.004*	*0.002*	*<0.001*	*<0.001*
**Body mass index**				
≤24,9	4.9 ± 3.9	5.2 ± 4.2	2.4 ± 2.7	12.4 ± 8.7
25–29,9	4.7 ± 3.1	4.6 ± 3.6	3.0 ± 2.3	12.2 ± 7.3
≥30	4.5 ± 3.1	4.7 ± 3.3	3.0 ± 2.2	12.2 ± 7.0
**p-values**	0.848	0.831	0.181	0.955

*Data are expressed as means ± standard deviation.

According to the IPAQ, the mean energy consumption per week was 1205.2 ± 1639.9 MET-min/week. There was a significant relationship between the level of physical activity and the mean scores of total and all MRS subscales. Women with low levels of physical activity had higher scores in all MRS domains than those with moderate and high levels of physical activity (Table 2). There were negative and significant correlations between total MRS scores (r = -0.205; p < 0.01), somato-vegetative (r = -0.131; p < 0.05), psychological (r = -0.176; p < 0.01) and urogenital (r = -0.189; P < 0.01) subscales’ scores and the physical activity levels of the women. These variables were also compared with demographic and health related factors (Table 3).

**Table 3:** Correlations between sociodemographic and health-related characteristics and total and subscale scores of MRS

		**1**	**2**	**3**	**4**	**5**	**6**	**7**	**8**	**9**	**10**	**11**	**12**	**13**	**14**
**1**	**Age**	-	-0.098	-0.052	*0.163*†	*0.217*†	**-***0.244*†	-0.060	*0.217*†	-0.004	0.074	-0.038	-0.044	0.013	-0.041
**2**	**Education**		-	*0.392*†	-0.090	0.088	-0.042	-0.017	**-***0.155*†	0.086	-0.159†	-0.121*	-0.105	-0.070	-0.126*
**3**	**Employment status**			-	-0.098	**-***0.150*†	-0.055	-0.052	-0.077	0.077	-0.153†	-0.150†	-0.104	-0.041	-0.126*
**4**	**Marital status**				-	0.009	0.000	0.036	-0.013	-0.032	0.025	0.020	0.062	*-0.313*†	-0.042
**5**	**Chronic disease**					-	0.019	0.096	*0.180*†	-0.130*	0.093	0.138*	*0.160*†	0.122*	*0.179*†
**6**	**Smoking status**						-	0.003	-0.083	*0.155*†	-0.071	0.097	0.084	0.006	0.092
**7**	**HRT**							-	0.147*	0.056	-0.084	0.114*	0.060	0.068	0.090
**8**	**BMI**								-	*-0.185*†	*0.119*†	0.049	0.035	0.111	0.074
**9**	**IPAQ**									-	*-0.347*†	-0.131*	*-0.176*†	*-0.189*†	*-0.205*†
**10**	**Sitting**										-	0.102	0.124*	0.090	0.126*
**11**	**Somato-vegetative**											-	*0.531*†	*0.368*†	*0.821*†
**12**	**Psychological**												**-**	*0.378*†	*0.837*†
**13**	**Urogenital**													-	*0.656*†
**14**	**Total MRS**														-

*p < 0.05 †p < 0.01.

The median reported sitting time was 360 minutes/day, with an interquartile range of 300–420 minutes. There were weak but significant correlations between the time spent sitting on a weekday in the last 7 days and BMI (r = 0.119; p < 0.01 ), total physical activity score(r = -0.347, p < 0.01), psychological subscale (r = 0.124, p < 0.05) and total MRS score(r = 0.126, p < 0.05) (Table 3).

Table 4 shows the frequency of the 11 symptoms assessed by the MRS according to physical activity level and body mass index. The three most frequently reported symptoms were: Physical and mental exhaustion (76.1%), sexual problems (71.8%), and hot flashes and sweating (70.5%). A significant decreasing trend in the rate of both the sleep problems (p = 0.009) and indices of joint pain (p = 0.001) was observed to correspond to the progression in the level of physical activity; same as the urogenital symptoms (Sexual problems p = 0.043, Dryness of vagina p = 0.016), with the exception of bladder problems. There were no other relationships in terms of other subscale scores and physical activity level (p > 0.05) (Table 4). In general terms, the present study did not find any significant relationship between BMI and total MRS and subscale scores (Tables 2 and 3).

**Table 4:** Frequency of menopausal symptoms as assessed by the MRS in total and according to physical activity level and body mass index

	**Physical activity level n (%)**^**a**^				**Body mass index n (%)**^**a**^			
**MRS**	**Low**	**Moderate**	**High**	**p-values**	**≤24,9**	**25-29,9**	**≥30**	**p-values**
**Somato-vegetative**								
Hot flashes, sweating (n = 215)	123 (71.1)	78 (67.8)	14 (82.4)	0.455	18 (66.7)	61 (72.6)	136 (70.1)	0.824
Heart discomfort (n = 129)	77 (44.5)	46 (40.0)	6 (35.3)	0.626	17 (63.0)	36 (42.9)	76 (39.2)	0.064
Sleep problems (n = 160)	103 (59.5)	52 (45.2)	5 (29.4)	*0.009*	13 (48.1)	51 (60.7)	96 (49.5)	0.203
Joint and muscular discomfort (n = 201)	129 (74.6)	65 (56.5)	7 (41.2)	*0.001*	15 (55.6)	50 (59.5)	136 (70.1)	0.115
**Psychological**								
Depressive mood (n = 202)	120 (69.4)	72 (62.6)	10 (58.8)	0.396	12 (44.4)	51 (60.7)	139 (71.6)	*0.009*
Irritability (n = 203)	121 (69.9)	75 (65.2)	7 (41.2)	0.052	17 (63.0)	55 (65.5)	131 (67.5)	0.868
Anxiety (n = 136)	85 (49.1)	47 (40.9)	4 (23.5)	0.076	12 (44.4)	35 (41.7)	89 (45.9)	0.810
Physical and mental exhaustion (n = 232)	138 (79.8)	83 (72.2)	11 (64.7)	0.177	21 (77.8)	68 (81.0)	143 (73.7)	0.420
**Urogenital**								
Sexual problems (n = 219)	134 (77.5)	74 (64.3)	11 (64.7)	*0.043*	15 (55.6)	61 (72.6)	143 (73.7)	0.142
Bladder problems (n = 116)	75 (43.4)	34 (29.6)	7 (41.2)	0.059	7 (25.9)	29 (34.5)	80 (41.2)	0.227
Dryness of vagina (n = 101)	66 (38.2)	34 (29.6)	1 (5.9)	*0.016*	5 (18.5)	35 (41.7)	61 (31.4)	0.060

^a^Column% for each row showing the number of women having symptoms.

There were no significant differences between BMI groups according to all MRS subscales except, depressive mood (p = 0.009) within the psychological symptoms. A significant increasing trend in the rate of depressive mood was observed from normal through overweight to obese participants (Table 4).

## Discussion

The MRS is a scale which is widely used to assess the symptoms of the menopause. The present study illustrates the fact that the mean MRS total and subscale scores were lower than in a multinational study [21]. In a previous study in Korea, it was determined that the scores obtained were lower than the mean scores of North and Latin America [27]. In our study, the results were higher than the mean scores of Europe, Latin America and North America [23]. This suggests that cultural differences affect the severity of menopausal symptoms. Dennerstein indicated that menopausal symptoms tend to vary from culture to culture. Women in North America and Europe were found more likely to experience severe climacteric symptoms compared to women in Asian countries [28]. One previous study has suggested that in Asian countries, women play a significant role in society with advancing age; they become eligible to participate in religious ceremonies and because they accept menopausal symptoms as inevitable changes which are to be experienced, they are thus able to adapt in a short time and with a positive manner [29].

Although studies show a decrease in menopausal symptoms going eastwards, in our study, menopausal symptoms were observed to be above the average of western countries. Some women consider changes during the climacteric period to be a problem and seek treatment, while others are relatively unaffected by any occurring changes and therefore do not consider them to be a problem. Uncu et al. suggested that women who approach the menopause as a pathological period have more menopausal complaints. With increasing levels of education, a more positive perception of menopause has been reported for Turkish women [30,31]. In this study, the fact that the menopausal symptom scores are higher than other studies may be due to the overall lower level of education of the participants.

There are many studies investigating the relationship between menopausal symptoms and the factors affecting these symptoms. Similarly, in this study, depending on socio-demographic and health-related variables, several variations of menopausal symptoms were reported. Menopausal symptoms were experienced less by women who were at least high school graduates, working, with no history of chronic disease and a high level of physical activity. Previous studies have indicated a negative relationship between menopausal symptoms and educational levels [32-34]. This could be because having accurate and reliable knowledge about the period of menopause is facilitated with the increase in the level of education.

The study indicated that somato-vegetative symptoms in working or retired women are fewer than those with no work experience [27,35,36]. There was a significant difference in favor of working women in terms of general adjustment, social adjustment, and self-actualization [37]. Thus, having more opportunities to prove themselves may have a positive effect on the psychological status of working women.

As indicated in a previous study among a cohort of postmenopausal women, although sexually inactive women scored significantly higher in another study, marital status was not associated with urogenital symptoms, [27,38]. In our study, fewer urogenital symptoms were observed in the sexually inactive women. However, taking into consideration the cultural constraints in Turkey, we should not overlook the fact that people might sometimes not answer questions accurately in order to avoid culturally determined negative perceptions.

In this study, as compatible with existing studies, menopausal symptoms were observed more commonly in women with a history of chronic disease [39,40]. In contrast to the substantial majority of published studies supporting the finding that active cigarette smoking increases the risk for vasomotor symptoms [11,41-44]; we did not find an increased number of menopausal symptoms in smokers. The present study found that the somato-vegetative scores of women who used HRT were significantly higher compared to those who did not. These subjects’ HRT requirements suggested that they already had severe symptoms. Similarly; it was found in a cohort of 202,638 postmenopausal women, past hormone therapy users who had discontinued treatment were more likely to have hot flushes and night sweats [45].

For women with menopausal symptoms who aim for healthy living in the long term, regular exercise and keeping BMI within the normal range are the necessary lifestyle change recommendations [46].

To our knowledge, exercise and physical activity have benefits for a healthy life but also offer conflicting evidence for roles in menopausal symptoms. This study found a significant decrease in the mean scores of all MRS domains of women as their physical activity increase. Recently, similar results were observed in a Brazilian study of 370 women aged 40–65 years [9].

The hypothesis that physical activity may protect against vasomotor symptoms is based on the effects on the hypothalamic β-endorphin system: lowered β-endorphin may be a mechanism underlying vasomotor pathogenesis, and physical activity elevates β-endorphin. An alternative theory is that physical activity could provoke vasomotor symptoms in women who have a narrowed thermo-neutral zone [11]. Physical activity increases body core temperature and might thus stimulate more hot flushes, especially when performed at high exercise intensities. In this study, no significant relationship between physical activity and vasomotor symptoms was observed. Because of the conflicting results, there is considerable need for intervention studies which can determine the effect of physical activity and assess the effect of exercise intensity on vasomotor symptoms.

The present study showed that the incidence of joint and muscular discomfort, sexual problems and vaginal dryness was higher in women with reported lower levels of physical activity. Some studies reported that fewer somatic symptoms were observed in physically active women compared with sedentary women [6,7,9,47,48]. Suling et al. found that women who were physically active were less likely to experience vaginal dryness and loss of sexual desire [14]. However, it is not clear how physical activity affects sexual health.

Knowledge about the influence of physical activity on psychological symptoms is limited to theories. Some published studies have reported that physical activity reduces psychological symptoms by causing an increase in the level of β-endorphin [14,49,50]. Previously, a cohort study in Australia reported that the psychological symptoms of the menopause had no association with physical activity [47].

Among middle aged women, a mid-to-low intensity of exercise participation is higher [51], whereas in the presence of high-intensity exercise β-endorphin is more likely to occur [50]. The relationship between physical activity and menopausal symptoms may also change depending on the intensity of the physical activity.

Referring to the relationship between BMI and vasomotor symptoms, some studies reported that higher BMI was associated with increased vasomotor symptoms; while others like ours, have shown no association [7,13,52,53]. A high BMI potentially implies a greater amount of adipose tissue, and this may alleviate the symptoms by converting adrenal androgens to estrogens. However, in this case, exercise may increase vasomotor symptoms by reducing adipose tissue.

In this study, our finding of little or no relationship between BMI and the menopausal symptoms may be associated with endogenous estrogen levels in women with different body size. In addition, fluctuations in estradiol level which are associated with weight gain may affect symptoms.

This is a cross-sectional study and it may not provide definitive information about cause-and-effect relationships. Therefore, as a limitation of our study, we can’t fully determine whether pre-existing physical activity and BMI indicate various menopausal symptoms.

## Conclusions

The menopause is a period of life, and therefore should not be viewed as a disease. However, the changes associated with the menopause affect women in different levels and this induces some women to seek treatment. Although HRT remains the most effective treatment for vasomotor symptoms, many women, based on recent evidence, no longer prefer hormone replacement therapy and are now seeking alternatives. Therefore, during this period, regular physical activity can be recommended as effective lifestyle change. In conclusion, the present study found that although somato-vegetative, psychological and urogenital symptoms were all alleviated by physical activity, they were not related to BMI. These results can be promising for women who do not want to use pharmacological agents in the treatment of menopausal symptoms. Further intervention studies are needed to investigate the effect of physical activity and BMI on menopausal symptoms.

## Abbreviations

BMI: Body mass index; MRS: Menopause rating scale; IPAQ: International physical activity questionnaire; E1: Estradiol; E2: Estrone; STRAW: Stages of reproductive aging workshop; WHO: World Health Organization; HRT: Hormone replacement therapy.

## Competing interests

The authors declare that they have no competing interests.

## Authors’ contributions

Study design: MNT, MK, DG; data collection: MNT; analysis and interpretation of the data: MNT, MK, DG; manuscript preparation: MNT, MK, DG; revised the manuscript: MK, DG. All authors read and approved final manuscript.

## Pre-publication history

The pre-publication history for this paper can be accessed here:

http://www.biomedcentral.com/1472-6874/14/38/prepub
